# Patient acceptance of telemedicine for breast cancer follow-up: a cross-sectional study from Eastern China

**DOI:** 10.3389/fonc.2026.1931347

**Published:** 2026-09-09

**Authors:** Minghua Cao, Qifa Gao, Qiliang Lou, Yuan Li, Yusong Zhang, Xichen Wang, Mingang Zhu, Weiqiang Huang

**Affiliations:** Department of Thyroid and Breast Surgery, The First People’s Hospital of Jiashan, Jiashan Hospital Affiliated of Jiaxing University, Jiaxing, China

**Keywords:** breast cancer, follow-up care, mobile health, patient acceptance, Telemedicine

## Abstract

**Introduction:**

Telemedicine offers a potential solution to the challenges of long-term follow-up for the growing population of breast cancer survivors. However, its acceptance and the factors influencing it among Chinese patients are not well understood.

**Methods:**

This cross-sectional study evaluated telemedicine acceptance and its associated factors among 812 breast cancer patients at a tertiary hospital in Eastern China (June 2026) using structured telephone interviews.

**Results:**

Overall, 72.3% of patients showed high acceptance, yet familiarity was low: only 27.7% had prior telemedicine use and 53.4% were familiar with it. Multivariate logistic regression revealed that unmarried/divorced/widowed status (OR = 0.32), unemployment (OR = 0.30), and higher risk concerns (OR = 0.44) were independently linked to lower acceptance (all P<0.05), while higher education (junior high: OR = 2.77; bachelor+: OR = 5.97), diagnosis duration >12 months (OR = 2.67), and greater perceived benefits (OR = 12.00) were associated with higher acceptance (all P<0.05). Moderation analysis showed technological barriers reduced acceptance, but their interactions with perceived benefits (P = 0.125) and risk concerns (P = 0.090) were not significant. Telephone consultation was the most preferred modality (62.4%), especially among older, less educated, and lower-income patients, whereas video consultation was favored by younger, more educated, and higher-income patients.

**Discussion:**

While acceptance of telemedicine is high among Eastern Chinese breast cancer patients, familiarity and experience remain limited. These findings may inform future approaches to telemedicine implementation, including patient education, attention to technological barriers, and flexible modality options, particularly for vulnerable subgroups.

## 1 Introduction

Breast cancer is the most common malignancy and a leading cause of cancer-related death among women worldwide (1). According to global cancer statistics, breast cancer has become one of the most frequently diagnosed cancers globally, with its disease burden continuing to rise and posing major challenges to public health systems (2). In China, the incidence of breast cancer has shown a sustained upward trend over the past decades. In 2020, an estimated 416,371 new cases were reported, accounting for nearly 20% of all female cancers in the country (3, 4). Despite advances in early screening techniques and comprehensive treatment strategies, the growing number of breast cancer survivors has significantly increased the strain on healthcare systems with respect to long-term follow-up and chronic disease management (5).

Standardized follow-up after initial breast cancer treatment is important for monitoring tumor recurrence, managing treatment-related adverse effects, and alleviating patients’ psychological distress (6). Previous studies have shown that systematic follow-up not only facilitates early detection of recurrence but also improves patients’ quality of life and long-term survival outcomes. However, traditional face-to-face follow-up models have notable limitations, including insufficient geographic accessibility, high time costs, heavy economic burdens, and overconcentration of medical resources in tertiary hospitals (7, 8). In China, this problem is particularly pronounced, as uneven distribution of medical resources and an incompletely mature tiered healthcare system further exacerbate patient burdens (9).

Telemedicine, defined as the provision of healthcare services across time and geographic distances using information and communication technologies, has emerged as an important means of improving healthcare accessibility (10). Its modalities include telephone follow-up, video consultation, text-based communication, and remote health monitoring platforms. During the COVID-19 pandemic, telemedicine was rapidly adopted worldwide to ensure continuity of cancer care while minimizing infection risk (11–13). Multiple studies and systematic reviews have shown that telemedicine is associated with high patient satisfaction in oncology follow-up, effectively reduces transportation and time costs, and can achieve quality of care comparable to traditional face-to-face follow-up in certain clinical contexts (14, 15).

Systematic reviews and meta-analyses indicate that telemedicine is generally safe and feasible in cancer care, with outcomes comparable to traditional face-to-face follow-up in follow-up management and symptom monitoring, although its clinical effectiveness varies significantly across cancer types, intervention formats, and patient characteristics (16, 17). In breast cancer, telemedicine is primarily used for postoperative follow-up, symptom management, and health education (18). From a screening perspective, telemedicine may also facilitate remote symptom and treatment-toxicity screening and referral triage, thereby helping identify patients who require timely in-person assessment (19, 20). However, telemedicine cannot replace physical examination and imaging assessment, and some patients may remain concerned about the quality of non-face-to-face care (18).

Despite its clear potential advantages, successful implementation of telemedicine depends heavily on patient acceptance and willingness to use it. Previous studies have identified several key factors influencing telemedicine adoption, including insufficient technological proficiency, concerns about the accuracy and safety of remote diagnosis and treatment, privacy protection issues, and limitations in device and network accessibility (21, 22). Drawing on the Technology Acceptance Model (TAM) and the Unified Theory of Acceptance and Use of Technology (UTAUT), patients’ evaluations of “perceived usefulness” and “perceived ease of use” are considered core psychological mechanisms affecting their willingness to use telemedicine.

In the Chinese context, the national government has incorporated telemedicine into the “Internet + Healthcare” strategic framework to promote its application in equalizing urban and rural healthcare services, particularly in rural and medically underserved areas (23). However, empirical research on acceptance of telemedicine follow-up among Chinese breast cancer patients remains limited. Existing studies have largely focused on short-term telemedicine use during COVID-19 (24, 25) or on chronic disease management (26), with insufficient attention to oncology patients, particularly the breast cancer population.

Furthermore, limited research has simultaneously integrated demographic characteristics, disease-related factors, cognitive–psychological factors (such as perceived benefits and risk concerns), and technological barriers in a single analytical framework. Therefore, it is important to identify factors associated with telemedicine acceptance among breast cancer patients in the Chinese context and to explore how demographic, clinical, psychosocial, and technological factors relate to acceptance.

This study aimed to assess the levels of familiarity, attitudes, concerns, and acceptance of telemedicine for follow-up and related health management services among breast cancer patients in Eastern China, and to further identify demographic, clinical, and psychosocial factors associated with willingness to use telemedicine. Specifically, perceived benefits were operationalized as perceived usefulness, while technological barriers reflected perceived ease of use; social structural factors (marital status, employment) were informed by UTAUT’s social influence dimension.

## 2 Methods

### 2.1 Study design and participants

A cross-sectional study was conducted between June 16 and 30, 2026, at Jiashan First People’s Hospital, a tertiary hospital in Zhejiang Province, Eastern China. A two-stage recruitment procedure was used. First, trained research assistants screened the hospital’s electronic medical record (EMR) system to identify female patients with histopathologically confirmed primary breast cancer who had attended outpatient or inpatient services during the preceding 12 months (July 2025 to June 2026). Second, the identified patients were contacted by telephone. During the call, research assistants verified the patient’s identity, reconfirmed the breast cancer diagnosis, explained the purpose and procedures of the study, and assessed eligibility using a standardized screening protocol. Inclusion criteria were: (1) Age ≥ 18 years (confirmed by ID); (2) Histopathologically confirmed primary breast cancer (confirmed via EMR); (3) Had completed active treatment (surgery, chemotherapy, radiotherapy) OR were currently receiving adjuvant endocrine or targeted therapy (i.e., not in the acute induction phase); (4) Had been formally informed of their cancer diagnosis by their primary physician (we asked patients directly: “Have you been told by your doctor that you have breast cancer?”). Exclusion criteria were: (1) *De novo* Stage IV (metastatic) disease; (2) Unable to complete the questionnaire due to cognitive impairment, severe hearing loss, or speech difficulties; (3) Acute severe comorbidities (e.g., stroke, acute myocardial infarction) that prevented them from participating in a 15–20 minutes telephone interview. Patients with *de novo* metastatic breast cancer at initial diagnosis were excluded; however, patients who progressed to stage IV during follow-up were not excluded.

A total of 1,043 potentially eligible patients were identified from the hospital database. Trained research assistants contacted them by telephone. After verifying eligibility and explaining the study, 127 patients declined participation and 104 were excluded (38 with cognitive/hearing impairment, 42 with *de novo* metastasis, 24 with severe comorbidities), yielding 812 valid interviews (effective response rate: 86.5% among eligible patients). Verbal informed consent was obtained from all participants at the start of the call.

### 2.2 Data collection

Data were collected through structured telephone interviews conducted by three uniformly trained research assistants. All interviewers used the same standardized questionnaire and followed consistent interview procedures. The questionnaire items were developed by reviewing previous literature on telemedicine acceptance and by mapping relevant constructs from the Technology Acceptance Model (TAM) and Health Belief Model (HBM) to the context of breast cancer follow-up. Items assessing perceived benefits were intended to capture patients’ perceptions of the usefulness and practical advantages of telemedicine, whereas risk-concern items addressed perceived safety and limitations of remote care. Technology-barrier items focused on device availability, digital literacy, and internet accessibility. The wording of items was contextually adapted to breast cancer follow-up and telephone-based administration. The complete questionnaire is provided in **Supplementary Appendix 1**.

The structured questionnaire comprised four sections. The first section collected demographic and clinical characteristics, including age, marital status, education level, occupation, monthly household income, place of residence, current province of residence, time since breast cancer diagnosis, cancer stage, and treatment completion status. The second section assessed familiarity with telemedicine. Participants rated their familiarity with the concept of telemedicine, including its application in breast cancer follow-up, on a 4-point scale ranging from 1 (not at all familiar) to 4 (very familiar). They were also asked whether they had previously used telemedicine services for any medical consultation. The third section assessed attitudes toward and concerns about telemedicine, including perceived benefits (e.g., reduced travel, cost savings, and avoidance of crowded clinics), risk concerns (e.g., concerns regarding assessment accuracy, potential medical errors, and lack of direct contact), and technology-related barriers (e.g., lack of appropriate devices, limited digital literacy, and poor internet access). Responses were recorded using a 4-point Likert scale ranging from 1 (strongly disagree) to 4 (strongly agree). Summary scores were calculated for perceived benefits, risk concerns, and technological barriers, with Cronbach’s α values of 0.87, 0.84, and 0.82, respectively. The fourth section assessed telemedicine acceptance. Participants rated their comfort with using telemedicine for breast cancer follow-up on a scale from 1 (not at all comfortable) to 10 (very comfortable). For the present analysis, scores of 1–5 were categorized as low acceptance, whereas scores of 6–10 were categorized as high acceptance. Participants were also asked to indicate their preferred telemedicine consultation modality, including telephone consultation, video consultation, text-based consultation, or other modalities.

Questionnaire completeness and response quality were checked by the interviewers during data collection. Before statistical analysis, the dataset was systematically screened for missing values, out-of-range values, logical inconsistencies, and duplicate records.

### 2.3 Statistical analysis

All statistical analyses were performed using SPSS 26.0 (IBM Corp., Armonk, NY, USA). All tests were two-sided, and the significance level was set at P < 0.05. Continuous variables were expressed as mean ± standard deviation, and categorical variables as frequencies and percentages. Based on the operational definition described above, participants were categorized into low-acceptance (1–5 points) and high-acceptance (6–10 points) groups. Between-group comparisons were performed using chi-square tests for categorical variables and independent-sample t-tests or one-way analysis of variance for continuous variables.

Variables with P < 0.05 in the univariable logistic regression analyses were then entered simultaneously into the multivariable logistic regression model using the enter method. Results were expressed as odds ratios (ORs) with 95% confidence intervals (CIs). Multicollinearity among predictors included in the multivariable logistic regression model was assessed using variance inflation factors (VIFs). VIF values >5 were considered indicative of potentially meaningful multicollinearity. Model fit was assessed using the Hosmer–Lemeshow goodness-of-fit test and Nagelkerke R², while model discrimination was evaluated using the area under the receiver operating characteristic curve (AUC). To further explore the potential moderating role of technological barriers, extended models including interaction terms (perceived benefits × technological barriers, risk concerns × technological barriers) were constructed. All continuous variables were mean- centered before constructing interaction terms to reduce multicollinearity. For significant interactions, simple slope analyses were performed, and predicted probability plots across different levels of technological barriers were generated to illustrate the direction and strength of the moderating effects.

As a sensitivity analysis, the original 1–10 telemedicine acceptance score was additionally analyzed as a continuous outcome using multivariable linear regression. Regression coefficients (β) and 95% confidence intervals (CIs) were reported. A prespecified theory-informed multivariable logistic regression model was additionally fitted by simultaneously adjusting for all demographic, clinical, telemedicine-related, and psychosocial variables considered conceptually relevant, irrespective of their univariable P values.

### 2.4 Ethics approval and informed consent

The study protocol was approved by the Institutional Review Board of Jiashan First People’s Hospital (Approval No. LW2026054). Given the minimal-risk and anonymous nature of the telephone survey, the IRB waived the requirement for written informed consent. Verbal informed consent was obtained from all participants before the interview. Participants were informed that participation was voluntary, that they could withdraw at any time without affecting their medical care, and that their responses would be kept confidential and used solely for research purposes. No directly identifiable personal information was retained in the final dataset.

## 3 Results

### 3.1 Baseline patient characteristics

A total of 812 breast cancer patients were included, of whom 225 (27.7%) were in the low-acceptance group and 587 (72.3%) in the high-acceptance group. Compared with the low-acceptance group, patients in the high-acceptance group were younger, had a higher proportion of married individuals, higher education levels, higher employment rates, higher income levels, and were more likely to reside in urban areas (all P < 0.001).

Regarding clinical characteristics, patients in the high-acceptance group were more likely to have a shorter diagnosis duration (6–12 months), whereas those in the low-acceptance group had a higher proportion of diagnosis duration >12 months (P = 0.020). Additionally, the proportion of those “unaware of stage” was significantly higher in the low-acceptance group (P < 0.001), and the two groups also differed significantly in treatment completion status (P < 0.001).

In terms of telemedicine-related characteristics, patients in the high-acceptance group had greater familiarity with telemedicine (62.0% vs. 31.1%, P < 0.001) and more prior usage experience (34.4% vs. 10.2%, P < 0.001).

In terms of psychological and cognitive dimensions, patients in the high-acceptance group had higher perceived benefit scores (3.27 ± 0.39 vs. 2.93 ± 0.32), lower risk concern scores (2.99 ± 0.41 vs. 3.09 ± 0.37), and lower technological barrier scores (1.96 ± 0.58 vs. 2.57 ± 0.52) (all P < 0.001). Regarding preferred telemedicine consultation modalities, telephone was the most preferred (62.4%), followed by video consultation (25.9%), text consultation (10.1%), and other modalities (1.6%). Detailed results are presented in **Table 1**.

**Table 1:** Baseline characteristics of breast cancer patients (N = 812).

Variables	All patients (N = 812)	Low acceptance (N = 225)	High acceptance (N = 587)	P value
Age, years (n, %)				<0.001
< 60	463 (57.0)	59 (26.2)	404 (68.8)	
≥ 60	349 (43.0)	166 (73.8)	183 (31.2)	
Marital status (n, %)				<0.001
Married	713 (87.8)	162 (72.0)	551 (93.9)	
Unmarried/Divorced/ Separated/Widowed	99 (12.2)	63 (28.0)	36 (6.1)	
Education level (n, %)				<0.001
Primary school and below	240 (29.6)	139 (61.8)	101 (17.2)	
Junior high school	315 (38.8)	65 (28.9)	250 (42.6)	
Senior high school	73 (9.0)	10 (4.4)	63 (10.7)	
College	68 (8.4)	8 (3.6)	60 (10.2)	
Bachelor’s degree and above	116 (14.2)	3 (1.3)	113 (19.3)	
Employment status (n, %)				<0.001
Employed	313 (38.5)	25 (11.1)	288 (49.1)	
Housewife	161 (19.8)	56 (24.9)	105 (17.9)	
Unemployed	88 (10.8)	55 (24.4)	33 (5.6)	
Retired	250 (30.9)	89 (39.6)	161 (27.4)	
Monthly household income, RMB (n, %)				<0.001
≤ 10,000	360 (44.3)	168 (74.7)	192 (32.7)	
> 10,000	452 (55.7)	57 (25.3)	395 (67.3)	
Residence (n, %)				<0.001
Urban	524 (64.5)	99 (44.0)	425 (72.4)	
Rural/Suburban	288 (35.5)	126 (56.0)	162 (27.6)	
Duration since breast cancer diagnosis (n, %)				0.020
< 6 months	103 (12.7)	35 (15.6)	68 (11.6)	
6–12 months	155 (19.1)	30 (13.3)	125 (21.3)	
> 12 months	554 (68.2)	160 (71.1)	394 (67.1)	
Cancer stage (n, %)				<0.001
Stage I	167 (20.6)	18 (8.0)	149 (25.4)	
Stage II	377 (46.4)	66 (29.3)	311 (53.0)	
Stage III	76 (9.4)	32 (14.2)	44 (7.5)	
Stage IV	8 (1.0)	4 (1.8)	4 (0.7)	
Unknown	184 (22.7)	105 (46.7)	79 (13.4)	
Treatment completion status (n, %)				<0.001
Not initiated	13 (1.6)	6 (2.7)	7 (1.2)	
Under treatment	611 (75.2)	144 (64.0)	467 (79.6)	
Completed < 1 year ago	42 (5.2)	12 (5.3)	30 (5.1)	
Completed ≥ 1 year ago	146 (18.0)	63 (28.0)	83 (14.1)	
Familiarity with telemedicine (n, %)				<0.001
Unfamiliar	378 (46.6)	155 (68.9)	223 (38.0)	
Familiar	434 (53.4)	70 (31.1)	364 (62.0)	
Prior telemedicine usage experience (n, %)				<0.001
No prior experience	587 (72.3)	202 (89.8)	385 (65.6)	
Has prior experience	225 (27.7)	23 (10.2)	202 (34.4)	
Preferred consultation mode (n, %)				<0.001
Phone call	507 (62.4)	174 (77.3)	333 (56.7)	
Video chat	210 (25.9)	26 (11.6)	184 (31.3)	
Text chat	82 (10.1)	15 (6.7)	67 (11.4)	
Others	13 (1.6)	10 (4.4)	3 (0.6)	
Perceived benefits of telemedicine, mean ± SD	3.17 ± 0.40	2.93 ± 0.32	3.27 ± 0.39	<0.001
Perceived risks of telemedicine, mean ± SD	3.02 ± 0.40	3.09 ± 0.37	2.99 ± 0.41	<0.001
Technical barriers to telemedicine, mean ± SD	2.13 ± 0.63	2.57 ± 0.52	1.96 ± 0.58	<0.001

### 3.2 Factors associated with telemedicine acceptance

As shown in **Table 2**, univariate analysis showed that age, marital status, education level, employment status, household income, place of residence, diagnosis duration, cancer stage, telemedicine familiarity, prior usage experience, perceived benefits, and risk concerns were significantly associated with telemedicine acceptance (all P < 0.05).

**Table 2:** Univariable and multivariate logistic regression analysis of factors associated with telemedicine acceptance among breast cancer patients.

Variables	Univariate analysis		Multivariable analysis*	
	OR (95%CI)	P value	OR (95%CI)	P value
Age				
< 60	Reference		Reference	
≥ 60	0.16 (0.11, 0.23)	< 0.001	0.63 (0.36, 1.08)	0.095
Marital status				
Married	Reference		Reference	
Unmarried/Divorced/ Separated/Widowed	0.17 (0.11, 0.26)	< 0.001	0.32 (0.17, 0.58)	< 0.001
Education level				
Primary school and below	Reference		Reference	
Junior high school	5.29 (3.66, 7.74)	< 0.001	2.77 (1.70, 4.54)	< 0.001
Senior high school	8.67 (4.42, 18.7)	< 0.001	2.64 (1.12, 6.64)	0.031
College	10.3 (4.99, 24.2)	< 0.001	2.46 (0.93, 7.03)	0.078
Bachelor’s degree and above	51.8 (18.8, 215)	< 0.001	5.97 (1.80, 27.40)	0.008
Employment status				
Employed	Reference		Reference	
Housewife	0.16 (0.10, 0.27)	< 0.001	0.67 (0.31, 1.42)	0.296
Unemployed	0.05 (0.03, 0.09)	< 0.001	0.30 (0.13, 0.70)	0.005
Retired	0.16 (0.10, 0.25)	< 0.001	0.60 (0.30, 1.19)	0.148
Monthly household income, RMB				
≤ 10,000	Reference		Reference	
> 10,000	6.06 (4.31, 8.63)	< 0.001	1.36 (0.82, 2.25)	0.231
Residence				
Urban	Reference		Reference	
Rural/Suburban	0.30 (0.22, 0.41)	< 0.001	0.84 (0.51, 1.40)	0.510
Duration since breast cancer diagnosis				
< 6 months	Reference		Reference	
6–12 months	2.14 (1.21, 3.81)	0.009	1.91 (0.90, 4.08)	0.094
> 12 months	1.27 (0.80, 1.97)	0.299	2.67 (1.38, 5.18)	0.004
Cancer stage				
Stage I	Reference		Reference	
Stage II	0.57 (0.32, 0.97)	0.047	1.25 (0.61, 2.47)	0.537
Stage III	0.17 (0.08, 0.32)	< 0.001	0.71 (0.30, 1.66)	0.424
Stage IV	0.12 (0.03, 0.55)	0.005	0.32 (0.06, 1.83)	0.196
Unknown	0.09 (0.05, 0.16)	< 0.001	0.71 (0.33, 1.51)	0.383
Treatment completion status				
Not initiated	Reference			
Under treatment	2.78 (0.92, 8.40)	0.070		
Completed < 1 year ago	2.14 (0.60, 7.70)	0.243		
Completed ≥ 1 year ago	1.13 (0.36, 3.53)	0.834		
Familiarity with telemedicine				
Unfamiliar	Reference		Reference	
Familiar	3.61 (2.60, 5.02)	< 0.001	1.10 (0.67, 1.81)	0.696
Prior telemedicine usage experience				
No prior experience	Reference		Reference	
Has prior experience	4.61 (2.95, 7.50)	< 0.001	1.51 (0.83, 2.80)	0.182
Perceived benefits of telemedicine	21.3 (11.5, 41.6)	< 0.001	12.00 (5.81, 24.81)	< 0.001
Perceived risks of telemedicine	0.53 (0.35, 0.78)	0.001	0.44 (0.24, 0.83)	0.011

*Adjusted for age, marital status, education level, employment status, monthly household income, residence, duration since breast cancer diagnosis, cancer stage, familiarity with telemedicine, prior telemedicine usage experience, perceived benefits of telemedicine, and risk concerns of telemedicine.

Multivariate logistic regression analysis revealed that, after adjusting for potential confounders, unmarried/divorced/separated/widowed status (OR = 0.32, 95% CI: 0.17–0.58), unemployment or being out of work (OR = 0.30, 95% CI: 0.13–0.70), and higher risk concerns (OR = 0.44, 95% CI: 0.24–0.83) were significantly associated with lower telemedicine acceptance.

In contrast, higher education level (junior high school: OR = 2.77; senior high school: OR = 2.64; bachelor’s degree or above: OR = 5.97), diagnosis duration >12 months (OR = 2.67), and higher perceived benefits (OR = 12.00, 95% CI: 5.81–24.81) were significantly associated with higher telemedicine acceptance.

After inclusion of covariates, the associations of age, household income, place of residence, cancer stage, telemedicine familiarity, and prior usage experience with acceptance were no longer statistically significant.

VIF values ranged from 1.0 to 2.1, indicating no substantial multicollinearity. The model showed adequate calibration (Hosmer–Lemeshow P = 0.130; Nagelkerke R² = 0.501) and good discrimination (AUC = 0.875, 95% CI: 0.848–0.901).

### 3.3 Moderating effect of technological barriers

**Figure 1:**
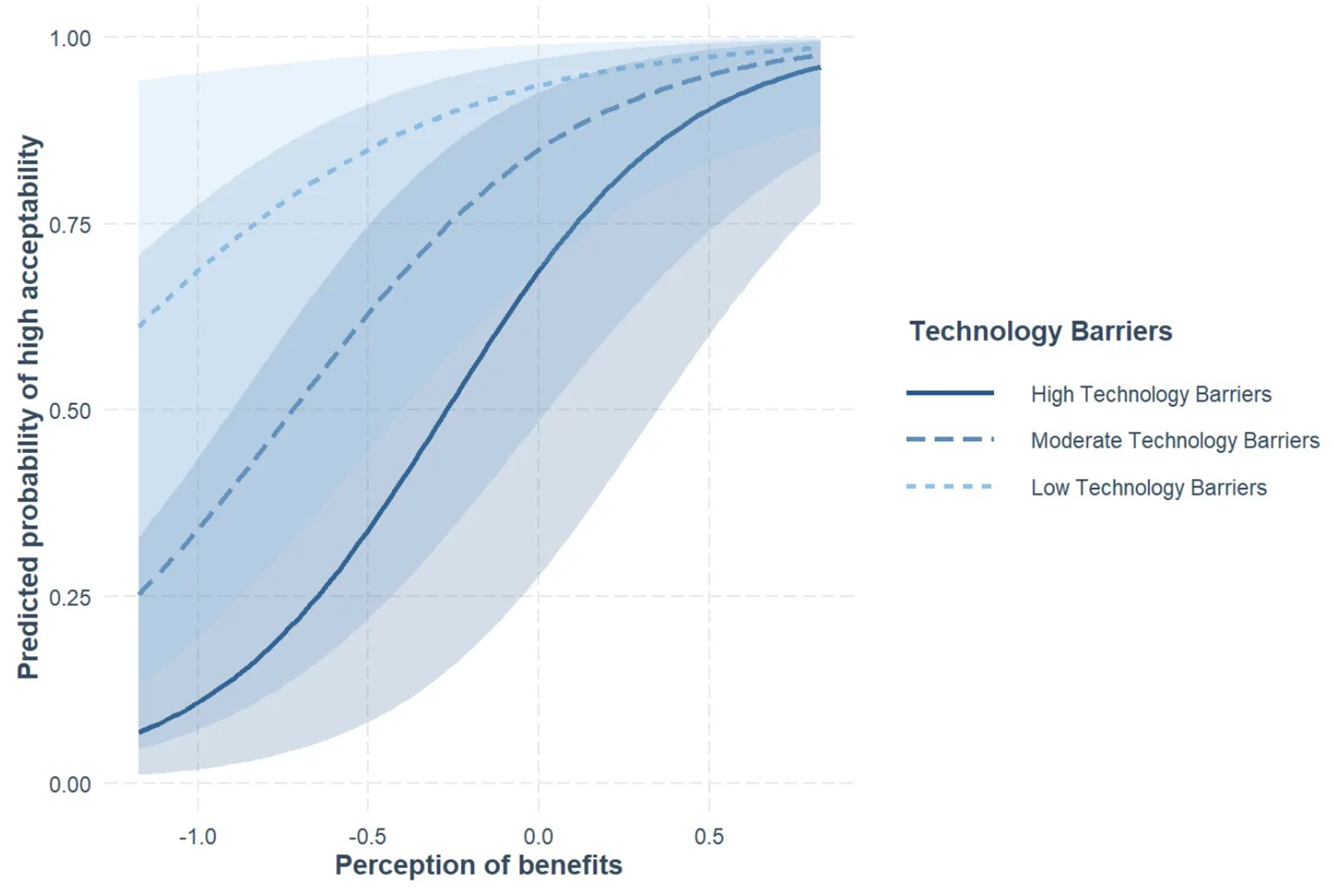
The moderating effect of telemedicine-related technological barriers on the association between perceived benefits and acceptance of telemedicine.

As shown in **Table 3**, in the extended model, perceived benefits of telemedicine were significantly positively associated with acceptance (OR = 10.62, 95% CI: 4.73–26.07), whereas technological barriers were significantly negatively associated with acceptance (OR = 0.23, 95% CI: 0.14–0.36) (both P < 0.001). However, the interaction term between perceived benefits and technological barriers did not reach statistical significance (OR = 2.19, P = 0.125). As shown in **Figure 1**, the relationship between perceived benefits and acceptance showed a consistently positive trend across different levels of technological barriers, but between-group differences were limited.

**Table 3:** Moderating effect of technical barriers to telemedicine on the association between perceived telemedicine benefits and telemedicine acceptance.

Variables	Adjusted model*	
	OR (95%CI)	P value
Perceived benefits of telemedicine	10.62 (4.73, 26.07)	< 0.001
Technical barriers to telemedicine	0.23 (0.14, 0.36)	< 0.001
Perceived telemedicine benefits × Technical barriers to telemedicine	2.19 (0.81, 6.06)	0.125

*Adjusted for age, marital status, education level, employment status, monthly household income, residence, duration since breast cancer diagnosis, cancer stage, familiarity with telemedicine, prior telemedicine usage experience, perceived benefits of telemedicine, risk concerns of telemedicine, technical barriers to telemedicine, and the interaction term between perceived telemedicine benefits and technical barriers to telemedicine.

**Figure 2:**
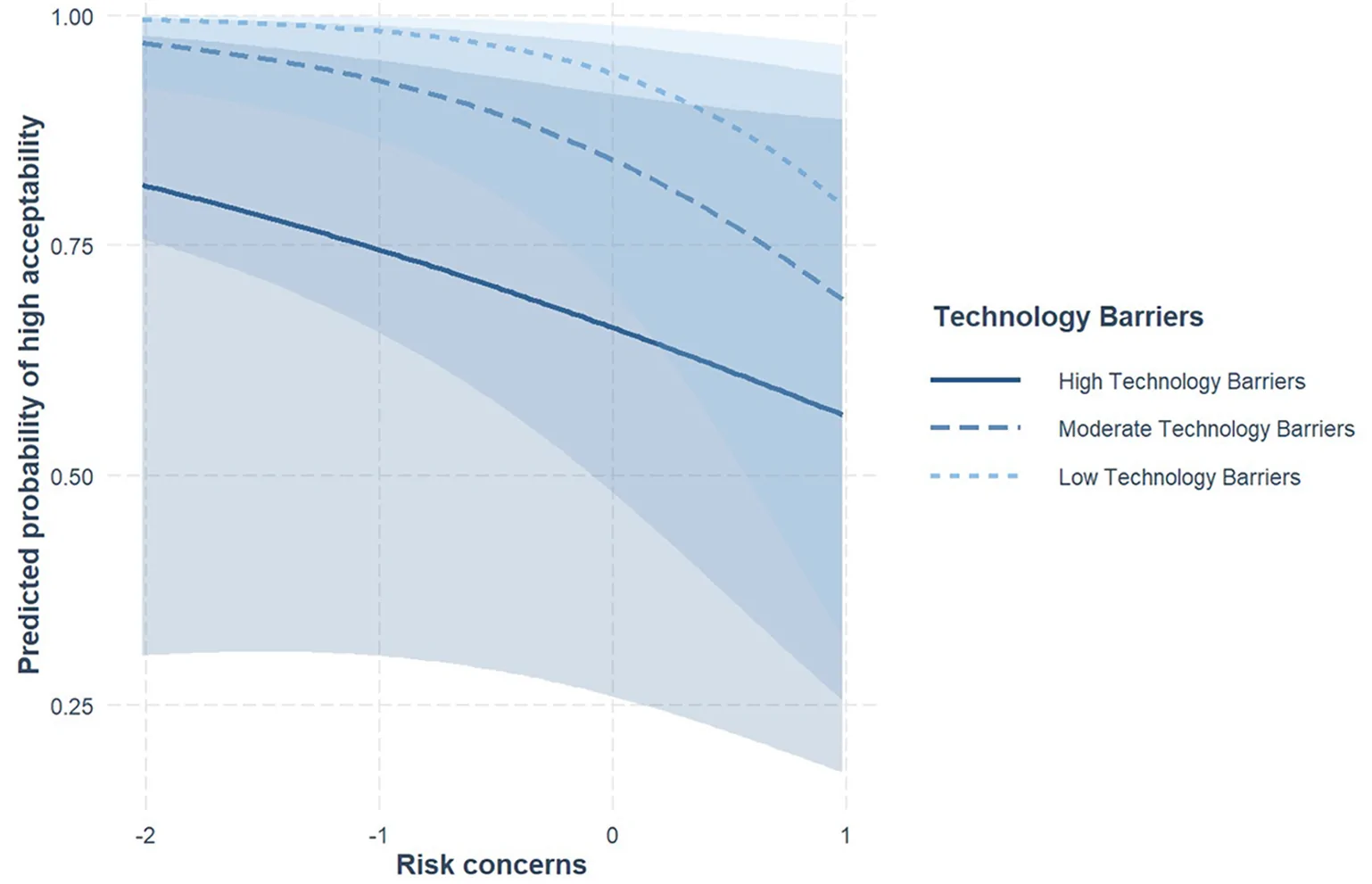
The moderating effect of telemedicine-related technological barriers on the association between risk concerns and acceptance of telemedicine.

As shown in **Table 4**, in the risk concern model, risk concerns were negatively associated with acceptance (OR = 0.40, 95% CI: 0.19–0.83), and technological barriers continued to show a significant negative effect (OR = 0.20, 95% CI: 0.12–0.32) (P < 0.05). The interaction term between risk concerns and technological barriers did not reach statistical significance (OR = 2.18, P = 0.090). As shown in **Figure 2**, the negative association between risk concerns and acceptance was consistent across different levels of technological barriers.

**Table 4:** Moderating effect of technical barriers to telemedicine on the association between perceived telemedicine risks and telemedicine acceptance.

Variables	Adjusted model*	
	OR (95%CI)	P value
Perceived risks of telemedicine	0.40 (0.19, 0.83)	0.015
Technical barriers to telemedicine	0.20 (0.12, 0.32)	< 0.001
Perceived telemedicine risks × Technical barriers to telemedicine	2.18 (0.90, 5.48)	0.090

*Adjusted for age, marital status, education level, employment status, monthly household income, residence, duration since breast cancer diagnosis, cancer stage, familiarity with telemedicine, prior telemedicine usage experience, perceived benefits of telemedicine, risk concerns of telemedicine, technical barriers to telemedicine, and the interaction term between perceived telemedicine risks and technical barriers to telemedicine.

### 3.4 Telemedicine modality preferences

As shown in **Table 5**, overall, telephone consultation was preferred by 507 participants (62.4%), followed by video consultation (25.9%), text-based consultation (10.1%), and other modalities (1.6%). Preferences differed across the examined subgroups (P < 0.05). Telephone consultation was particularly common among participants aged ≥60 years (80.8%), those with primary-school education or below (82.5%), unemployed participants (81.7%), and those with lower household income (75.8%). In contrast, video consultation was more frequently preferred by participants aged <60 years (35.2%) and was the leading choice among those with a bachelor’s degree or above (52.6%).

**Table 5:** Comparison of preferred telemedicine consultation modes across subgroups.

Variables	Phone call	Video chat	Text chat	Others	P value
Age					< 0.001
< 60	225 (48.6)	163 (35.2)	72 (15.6)	3 (0.6)	
≥ 60	282 (80.8)	47 (13.4)	10 (2.9)	10 (2.9)	
Marital status					0.017
Married	433 (60.7)	195 (27.3)	75 (10.5)	10 (1.4)	
Unmarried/Divorced/Separated/Widowed	74 (74.7)	15 (15.2)	7 (7.1)	3 (3.0)	
Education level					< 0.001
Primary school and below	198 (82.5)	23 (9.6)	12 (5.0)	7 (2.9)	
Junior high school	204 (64.8)	76 (24.1)	31 (9.8)	4 (1.3)	
Senior high school	34 (46.6)	22 (30.1)	15 (20.5)	2 (2.7)	
College	27 (39.7)	28 (41.2)	13 (19.1)	0 (0.0)	
Bachelor’s degree and above	44 (37.9)	61 (52.6)	11 (9.5)	0 (0.0)	
Employment status					< 0.001
Employed	136 (43.5)	125 (39.9)	51 (16.3)	1 (0.3)	
Housewife	114 (70.8)	28 (17.4)	12 (7.5)	7 (4.3)	
Unemployed	72 (81.7)	10 (11.4)	5 (5.7)	1 (1.1)	
Retired	185 (74.0)	47 (18.8)	14 (5.6)	4 (1.6)	
Monthly household income, RMB					< 0.001
≤ 10,000	273 (75.8)	54 (15.0)	24 (6.7)	9 (2.5)	
> 10,000	234 (51.8)	156 (34.5)	58 (12.8)	4 (0.9)	
Residence					< 0.001
Urban	305 (58.2)	158 (30.2)	59 (11.3)	2 (0.4)	
Rural/Suburban	202 (70.1)	52 (18.1)	23 (8.0)	11 (3.8)	
Duration since breast cancer diagnosis					< 0.001
< 6 months	40 (38.8)	45 (43.7)	15 (14.6)	3 (2.9)	
6–12 months	81 (52.3)	43 (27.7)	30 (19.4)	1 (0.6)	
> 12 months	386 (69.7)	122 (22.0)	37 (6.7)	9 (1.6)	
Cancer stage					< 0.001
Stage I	60 (35.9)	80 (47.9)	26 (15.6)	1 (0.6)	
Stage II	241 (63.9)	99 (26.3)	37 (9.8)	0 (0.0)	
Stage III	52 (68.4)	14 (18.4)	9 (11.8)	1 (0.6)	
Stage IV	6 (75.0)	2 (25.0)	0 (0.0)	0 (0.0)	
Unknown	148 (80.4)	15 (8.2)	10 (5.4)	11 (6.0)	
Treatment completion status					< 0.001
Not initiated	5 (38.5)	4 (30.8)	2 (15.4)	2 (15.4)	
Under treatment	365 (59.7)	178 (29.1)	66 (10.8)	2 (0.3)	
Completed < 1 year ago	27 (64.3)	11 (26.2)	3 (7.1)	1 (2.4)	
Completed ≥ 1 year ago	110 (75.3)	17 (11.6)	11 (7.5)	8 (5.5)	
Familiarity with telemedicine					< 0.001
Unfamiliar	279 (73.8)	61 (16.1)	26 (6.9)	12 (3.2)	
Familiar	228 (52.5)	149 (34.3)	56 (12.9)	1 (0.2)	
Prior telemedicine usage experience					< 0.001
No prior experience	411 (70.0)	118 (20.1)	46 (7.8)	12 (2.0)	
Has prior experience	96 (42.7)	92 (40.9)	36 (16.0)	1 (0.4)	
Perceived benefits of telemedicine, mean ± SD	3.12 ± 0.38	3.29 ± 0.42	3.27 ± 0.44	2.83 ± 0.44	< 0.001
Perceived risks of telemedicine, mean ± SD	3.06 ± 0.38	2.97 ± 0.38	2.88 ± 0.55	3.04 ± 0.22	< 0.001

### 3.5 Sensitivity analysis

The original telemedicine acceptance score ranged from 1 to 10, with a mean of 6.50 ± 2.44. As shown in **Supplementary Table 1**, in the sensitivity analysis treating the original acceptance score as a continuous outcome, higher perceived benefits remained strongly and positively associated with telemedicine acceptance (β = 1.82, 95% CI: 1.45–2.12, P < 0.001). Older age (≥60 years; β = −0.88, 95% CI: −1.26 to −0.53, P < 0.001), being unmarried/divorced/separated/widowed (β = −0.99, 95% CI: −1.40 to −0.62, P < 0.001), and unemployment (β = −0.93, 95% CI: −1.46 to −0.42, P < 0.001) remained negatively associated with acceptance, whereas higher educational attainment was positively associated with acceptance. Prior telemedicine experience, familiarity with telemedicine, household income, residence, cancer stage, treatment completion status, and perceived risks were not statistically significant in the adjusted model. Overall, the direction of the major associations was broadly consistent with the primary binary logistic regression analysis, although the magnitude and statistical significance of some individual associations differed between the two models.

As shown in **Supplementary Table 2**, sensitivity analysis using a theory-informed covariate set yielded results consistent with the primary analysis. The associations of marital status, education level, employment status, duration since breast cancer diagnosis, perceived benefits of telemedicine, and Perceived risks of telemedicine remained in the same direction and retained their statistical significance, whereas variables that were not significant in the primary model remained non-significant after full adjustment.

## 4 Discussion

This study found that, despite limited familiarity with and previous use of telemedicine, 72.3% of the participants had high telemedicine acceptance. Education level, duration since breast cancer diagnosis, perceived benefits, and risk concerns were independently associated with telemedicine acceptance. In the extended models, technological barriers were associated with lower acceptance, whereas their interactions with perceived benefits and risk concerns were not statistically significant. Overall, the findings suggest that telemedicine acceptance is associated with multiple domains, including cognitive appraisal, social structural characteristics, and technological accessibility.

### 4.1 Acceptance level and comparison with previous studies

Our finding that 72.3% of breast cancer patients had high telemedicine acceptance indicates that most patients are willing to adopt telemedicine as a supplementary approach for breast cancer follow-up and related health management services. This result is broadly consistent with acceptance rates of 65%–80% reported in international studies, suggesting a favorable patient foundation for telemedicine promotion among Chinese breast cancer patients. Additionally, our results align with studies from other countries indicating that telemedicine acceptance varies considerably by patient demographic characteristics, prior usage experience, disease stage, and healthcare service context (27–29). Previous systematic reviews on telemedicine have also indicated that telemedicine is generally feasible and acceptable, but its effectiveness is highly dependent on the specific disease context, service model, and patient characteristics (16, 17).

Notably, despite relatively high overall acceptance, patients’ actual exposure to telemedicine remained insufficient. Only 27.7% of patients in this study had prior telemedicine experience, suggesting that high willingness does not equate to adequate understanding or actual use. Similar gaps in digital healthcare implementation have been linked to limited digital literacy, inadequate access to devices or reliable internet, uncertainty regarding service pathways, and concerns about communication and care quality (12, 21). Although telemedicine use expanded rapidly during the COVID-19 pandemic (30), its sustainable integration into routine oncology care requires patient education, standardized workflows, technological support, and institutional commitment (12, 15).

### 4.2 Factors associated with telemedicine acceptance

Perceived benefits were strongly and positively associated with telemedicine acceptance in this study (OR = 12.00, 95% CI: 5.81–24.81, P < 0.001). International studies similarly show that patients with cancer value telemedicine because it can reduce travel, waiting time, financial burden, and disruption to daily life (29, 31, 32). This finding is also consistent with the Health Belief Model (33) and the concept of perceived usefulness in technology acceptance theory. For patients requiring long-term breast cancer follow-up, these practical benefits may make remote services more acceptable. These findings suggest that future telemedicine implementation could consider communicating concrete practical benefits, such as reduced travel and waiting time, while clearly explaining which clinical situations are appropriate for remote versus in-person care. The effectiveness of such approaches should be evaluated in future intervention studies.

Conversely, risk concerns were significantly negatively associated with telemedicine acceptance. Patient concerns about the accuracy of remote assessments, risks of medical errors, privacy and security, and lack of direct patient–provider contact may undermine their willingness to use telemedicine. Breast cancer follow-up encompasses multiple aspects including recurrence monitoring, adverse effect management, endocrine therapy adherence, psychological support, and interpretation of imaging findings; patients may worry that remote modalities cannot adequately substitute for face-to-face detailed history-taking and physical examination. Previous studies have also suggested that telemedicine acceptance depends not only on convenience but also on patient trust in care quality, safety, and adequacy of communication (27–29). Therefore, telemedicine in breast cancer management may be better suited as a stratified follow-up tool rather than a complete replacement for in-person care. For stable follow-up, result reporting, medication consultation, and health education, telemedicine has good applicability; face-to-face assessment may remain preferable in situations involving new symptoms, suspected recurrence, complex treatment decisions, or the need for physical examination.

Social structural characteristics were also associated with telemedicine acceptance. Unmarried/divorced/separated/widowed status and unemployment were associated with lower acceptance, consistent with previous findings that individuals with less social support and fewer economic resources face greater barriers to adopting new technologies (34, 35). A possible explanation is that patients lacking stable partner or family support may have greater difficulty obtaining assistance with device operation, information comprehension, or care decisions when encountering new service formats; those who are unemployed or economically disadvantaged may be more concerned about potential costs, device requirements, or network conditions. Although telemedicine theoretically improves accessibility, if platform design is complex, payment policies are unclear, or auxiliary support is lacking, it may paradoxically further restrict vulnerable populations. These findings suggest that future implementation strategies may need to consider process simplification, telephone follow-up options, caregiver involvement, and on-site guidance for patients who may experience greater barriers to telemedicine use. The effectiveness of these approaches remains to be evaluated prospectively.

Higher education level was consistently associated with higher telemedicine acceptance, consistent with other studies showing that more educated patients generally have better information comprehension, digital health literacy, and more positive attitudes toward novel healthcare models (36). Digital health literacy is considered an important capacity affecting whether patients can effectively access, understand, and use online health information (37). In China, although smartphones and internet applications are highly prevalent, a significant digital divide persists across age, education, and socioeconomic groups. These findings suggest that future implementation could consider lowering operational thresholds, improving information clarity, and providing accessible and actionable educational materials for patients with lower educational attainment.

We also found that patients with a diagnosis duration >12 months were more likely to accept telemedicine. This finding should be interpreted cautiously because the association between time since diagnosis and telemedicine acceptance has not been consistently established. One possible explanation is that patients with longer disease experience are more familiar with follow-up procedures and may place greater value on convenience. By contrast, an international oncology survey found that patients strongly preferred in-person care for the first postdiagnosis consultation and other sensitive discussions (31). Telemedicine may therefore be more acceptable for stable follow-up than for initial diagnosis, major treatment decisions, or emotionally complex consultations.

### 4.3 Moderating effect of technological barriers

This study showed that technological barriers were significantly negatively associated with telemedicine acceptance, indicating that internet access, device acquisition, platform operability, and confidence in using digital technologies remain important obstacles affecting patient acceptance. Previous systematic reviews on telemedicine adoption barriers have also identified inadequate technological infrastructure, limited user digital capacity, privacy and security concerns, and insufficient organizational support as common limiting factors (21, 38). For breast cancer patients, particularly those who are older, less educated, or lack family support, technological barriers may directly reduce their accessibility to and confidence in telemedicine.

However, we did not observe significant moderating effects of technological barriers on the relationships between perceived benefits or risk concerns and telemedicine acceptance. That is, although technological barriers themselves reduced acceptance, they did not significantly alter the strength of the associations between perceived benefits and acceptance or between risk concerns and acceptance. The interaction plots showed some differences in predicted probabilities across levels of technological barriers, but the interaction terms did not reach statistical significance. Therefore, it is inappropriate to characterize technological barriers as a clear moderator; rather, they are better interpreted as an independent impeding factor.

Several reasons may explain this result. First, the effect of technological barriers on telemedicine acceptance may be more of a “direct threshold effect.” When patients lack devices, network connectivity, or operational skills, even if they recognize the convenience of telemedicine, they may experience greater difficulty using telemedicine, which could partly explain the observed association between technological barriers and lower acceptance. Second, perceived benefits, risk concerns, and technological barriers may represent different levels of decision-making factors: perceived benefits and risk concerns reflect patients’ subjective evaluations of telemedicine’s value and safety, whereas technological barriers reflect usage conditions and capacity constraints. They may influence acceptance in parallel, without necessarily exhibiting significant interaction. Third, our dichotomization of telemedicine acceptance may have reduced statistical power to detect interaction effects. Future studies could adopt longitudinal designs, continuous acceptance scores, or structural equation modeling to further explore the pathways through which technological barriers affect telemedicine adoption.

### 4.4 Modality preferences and subgroup differences

This study found that telephone was the most preferred telemedicine modality overall (62.4%), consistent with previous studies indicating that telephone consultation has advantages in accessibility, familiarity, simple operation, and independence from complex digital skills (39). For breast cancer patients requiring long-term follow-up, telephone consultation may be a practical option for lower-complexity follow-up scenarios, although its clinical suitability should be determined according to the patient’s clinical status and the requirements of the consultation.

At the same time, we found significant differences in modality preferences across subgroups. Younger, more educated, higher-income, and employed participants more frequently preferred video or text consultation, whereas older and socioeconomically disadvantaged participants more frequently preferred telephone consultation. Similar disparities in the use of video versus audio-only telehealth have been reported in cancer populations in the United States and other settings (40, 41).

These subgroup differences suggest that a one-size-fits-all telemedicine approach may not adequately reflect the differing preferences and needs of patients. A multimodal service model that allows patients to choose among telephone, video, and text-based consultation may therefore be a potential direction for future implementation.

### 4.5 Clinical and policy implications

Our findings provide several considerations for future implementation of telemedicine in breast cancer follow-up. Patients may benefit from clear information regarding available modalities, procedures, potential benefits, and limitations, although the effectiveness of specific educational approaches requires further evaluation. Stable follow-up, medication counseling, result interpretation, and health education may be appropriate contexts for remote care, whereas new symptoms, suspected recurrence, or major treatment changes may require in-person assessment according to clinical judgment (12, 15, 29).

Future implementation strategies may also need to consider differences in patients’ digital capacities. Telephone follow-up, simplified interfaces, appointment assistance, and caregiver involvement could be considered for patients with greater digital access barriers (40), while the feasibility and effectiveness of these approaches should be evaluated prospectively. At the policy level, our findings may inform future discussions regarding reimbursement, quality standards, information-security safeguards, and referral pathways between remote and in-person care. However, the effectiveness, cost-effectiveness, and equity implications of specific policy approaches require evaluation in future implementation studies.

### 4.6 Strengths and limitations

This study has several strengths. First, the large sample size enabled systematic assessment of breast cancer patients’ familiarity, prior experience, perceived benefits, risk concerns, technological barriers, and acceptance of telemedicine, providing comprehensive empirical evidence for telemedicine service design for Chinese breast cancer patients. Second, grounded in the Health Belief Model and technology acceptance theories, our study incorporated multiple dimensions including cognitive factors, social structural factors, and technological barriers, providing a more comprehensive assessment of factors associated with telemedicine acceptance. Third, we further constructed moderation models to explore whether technological barriers modified the relationships between perceived benefits or risk concerns and acceptance, offering new hypotheses and references for future mechanistic research.

This study also has several limitations. First, the cross-sectional design precludes causal inference and cannot determine whether patient acceptance changes with increasing telemedicine experience. Second, the study was conducted at a single tertiary hospital in Eastern China, where regional economic development, medical resources, and internet infrastructure are relatively advanced; generalization of findings to Western China, primary care settings, or rural areas should be made with caution. Third, reliance on self-reported questionnaires may introduce recall and social desirability biases. Fourth, data were collected through telephone interviews, and patients with severe hearing impairment were excluded because they could not reliably complete the interview. Because telephone consultation was also the most commonly preferred telemedicine modality in our sample, the interview mode itself may have influenced participants’ responses and may have introduced selection or mode-related bias. Patients who were more comfortable with telephone-based communication may have been more likely to participate or may have reported greater preference for telephone consultation. Therefore, the observed preference for telephone consultation should be interpreted cautiously. Fifth, this study focused primarily on the patient side of acceptance and did not incorporate provider-side factors such as healthcare professional attitudes, platform usability, payment mechanisms, or institutional management processes. Finally, the study-specific questionnaire, although theoretically informed and demonstrating acceptable internal consistency, did not undergo formal expert-panel review, pilot testing, or other formal assessments of construct validity. Therefore, the extent to which the questionnaire fully captures the intended constructs warrants further evaluation in future studies.

## 5 Conclusion

This study demonstrates that breast cancer patients in Eastern China have high acceptance of telemedicine for follow-up, although their actual usage experience remains limited. Higher perceived benefits and higher education level were associated with greater telemedicine acceptance, whereas risk concerns, unmarried/divorced/separated/widowed status, and unemployment were associated with lower acceptance. Technological barriers are significantly associated with lower telemedicine acceptance, but no significant moderating effects were observed on the relationships between perceived benefits or risk concerns and acceptance. Telephone consultation was the most frequently preferred modality, particularly among older, less educated, and lower socioeconomic-status patients.

These findings may inform future patient-centered telemedicine implementation strategies, including attention to perceived benefits, technological barriers, and flexible modality options, particularly for vulnerable subgroups.

## Data Availability

The original contributions presented in the study are included in the article/**Supplementary Material**. Further inquiries can be directed to the corresponding authors.
