# Supplementary material for: Patient acceptance of telemedicine for breast cancer follow-up: a cross-sectional study from Eastern China
Source: Front Oncol. 2026 Sep 9;16:1931347. doi: 10.3389/fonc.2026.1931347 (PMC13597228; doi:10.3389/fonc.2026.1931347)
Supplement: Supplementary file 1 [file SupplementaryFile1.docx]

Questionnaire on Patients' Perceptions and Acceptance of Telemedicine for Breast Cancer Screening and Follow‑Up

Section A: Patient Demographic and Clinical Characteristics

1. Age: ______ years

2. Marital status

○ Single　○ Married　○ Divorced / Separated / Widowed

3. Ethnicity

○ Han Chinese　○ Other

4. Highest education level

○ No formal education　○ Primary school　○ Junior high school　○ Senior high school / Technical secondary school　○ Junior college　○ Bachelor's degree　○ Master's / Doctoral degree

5. Employment status

○ Employed　○ Homemaker　○ Unemployed / Out of work　○ Retired

6. Average monthly household income (RMB)

○ ≤ 5,000　○ 5,001–10,000　○ 10,001–15,000　○ 15,001–20,000

○ 20,001–25,000　○ 25,001–30,000　○ 30,001–35,000　○ 35,001–40,000

○ 40,001–45,000　○ 45,001–50,000　○ > 50,000

7. Place of residence

○ Urban　○ Suburban　○ Rural

8. Province of current residence/work: ______

9. Time since breast cancer diagnosis

○ < 6 months　○ 6–12 months ago　○ > 12 months

10. Cancer stage

○ Stage I　○ Stage II　○ Stage III　○ Stage IV　○ Unclear / Uncertain

11. Treatment completion status

○ Not yet started treatment　○ Currently undergoing treatment　○ Completed < 12 months ago　○ Completed ≥ 12 months ago

Section B: Familiarity with Telemedicine

12. How familiar are you with the concept of telemedicine for disease consultation, including breast cancer follow‑up?

○ Not at all familiar　○ Slightly familiar　○ Moderately familiar　○ Very familiar

13. Have you ever used telemedicine services for any medical consultation?

○ Yes　○ No

Section C: Attitudes

C1: Attitudes toward the use of telemedicine in breast cancer follow‑up

14. The use of telemedicine in breast cancer follow‑up is a feasible alternative that reduces the inconvenience of in‑person outpatient visits.

○ Strongly agree　○ Agree　○ Disagree　○ Strongly disagree

15. The use of telemedicine in breast cancer follow‑up is a feasible alternative that helps avoid missing in‑person appointments.

○ Strongly agree　○ Agree　○ Disagree　○ Strongly disagree

16. Automated alerts or reminders for breast cancer follow‑up via telemedicine can serve as useful prompts to help patients avoid missing routine screening.

○ Strongly agree　○ Agree　○ Disagree　○ Strongly disagree

17. Telemedicine for breast cancer follow‑up enables healthcare providers to deliver care to patients in remote areas.

○ Strongly agree　○ Agree　○ Disagree　○ Strongly disagree

18. Telemedicine for breast cancer follow‑up can avoid hospital visits for non‑critical breast cancer‑related issues.

○ Strongly agree　○ Agree　○ Disagree　○ Strongly disagree

19. Compared with in‑person outpatient follow‑up, telemedicine for breast cancer follow‑up can reduce costs.

○ Strongly agree　○ Agree　○ Disagree　○ Strongly disagree

20. Telemedicine for breast cancer follow‑up helps alleviate the stress caused by outpatient crowding or long waiting times.

○ Strongly agree　○ Agree　○ Disagree　○ Strongly disagree

21. Telemedicine for breast cancer follow‑up can overcome the embarrassment associated with physical breast examination.

○ Strongly agree　○ Agree　○ Disagree　○ Strongly disagree

22. Telemedicine for breast cancer follow‑up can alleviate hospital‑related anxiety (i.e., intense fear or anxiety about hospitalization).

○ Strongly agree　○ Agree　○ Disagree　○ Strongly disagree

23. Telemedicine for breast cancer follow‑up is necessary only during public health emergencies (e.g., pandemics, infectious disease outbreaks, floods, etc.).

○ Strongly agree　○ Agree　○ Disagree　○ Strongly disagree

C2: Concerns about the use of telemedicine in breast cancer follow‑up

24. The use of telemedicine services for breast cancer follow‑up is a concern, particularly regarding the accuracy of assessment compared with in‑person examination.

○ Strongly agree　○ Agree　○ Disagree　○ Strongly disagree

25. The absence of traditional face‑to‑face consultation in telemedicine may increase the risk of medical errors.

○ Strongly agree　○ Agree　○ Disagree　○ Strongly disagree

26. The use of telemedicine for breast cancer follow‑up is concerning, especially due to the lack of direct contact with the physician.

○ Strongly agree　○ Agree　○ Disagree　○ Strongly disagree

27. Telemedicine services for breast cancer follow‑up limit the ability to perform physical breast examination.

○ Strongly agree　○ Agree　○ Disagree　○ Strongly disagree

28. I have potential concerns about using telemedicine for breast cancer follow‑up due to lack of access to a computer, smartphone, or tablet.

○ Strongly agree　○ Agree　○ Disagree　○ Strongly disagree

29. I may experience difficulties in accessing telemedicine services for breast cancer follow‑up due to limited digital literacy or familiarity with digital tools.

○ Strongly agree　○ Agree　○ Disagree　○ Strongly disagree

30. Poor or limited internet service in my area may hinder my access to telemedicine services for breast cancer follow‑up.

○ Strongly agree　○ Agree　○ Disagree　○ Strongly disagree

Section D: Acceptability

31. On a scale from 1 to 10, where 1 represents "not at all comfortable" and 10 represents "very comfortable," please rate your comfort level with using telemedicine for breast cancer follow‑up:

1 (not at all comfortable) – 2 – 3 – 4 – 5 – 6 – 7 – 8 – 9 – 10 (very comfortable)

If your healthcare provider recommended telemedicine for your breast cancer follow‑up, how likely would you be to choose it?

○ Very unlikely　○ Unlikely　○ Somewhat likely　○ Very likely

32. What is your most preferred modality for telemedicine consultation?

○ Telephone call　○ Video chat　○ Text chat　○ Email　○ Other (please specify): ______
