# Supplementary material for: Patient acceptance of telemedicine for breast cancer follow-up: a cross-sectional study from Eastern China
Source: Front Oncol. 2026 Sep 9;16:1931347. doi: 10.3389/fonc.2026.1931347 (PMC13597228; doi:10.3389/fonc.2026.1931347)
Supplement: Supplementary file 2 [file Table1.docx]

**Supplemental Table S1.** Sensitivity analysis using telemedicine acceptance as a continuous outcome

| Variables | Crude model | | Adjusted model* | |
| --- | --- | --- | --- | --- |
|  | β (95%CI) | *P* value | β (95%CI) | *P* value |
| Age |  |  |  |  |
| < 60 | Reference |  | Reference |  |
| ≥ 60 | -2.53 (-2.82, -2.24) | < 0.001 | -0.88 (-1.26, -0.53) | < 0.001 |
| Marital status |  |  |  |  |
| Married | Reference |  | Reference |  |
| Unmarried/Divorced/  Separated/Widowed | -2.41 (-2.90, -1.92) | < 0.001 | -0.99 (-1.40, -0.62) | < 0.001 |
| Education level |  |  |  |  |
| Primary school and below | Reference |  | Reference |  |
| Junior high school | 2.06 (1.71, 4.92) | < 0.001 | 0.86 (0.51, 1.20) | < 0.001 |
| Senior high school | 2.94 (2.40, 3.48) | < 0.001 | 1.11 (0.56, 1.20) | < 0.001 |
| College | 2.98 (2.43, 3.54) | < 0.001 | 0.70 (0.12, 1.23) | 0.013 |
| Bachelor’s degree and above | 3.71 (3.25, 4.16) | < 0.001 | 1.05 (0.44, 1.52) | < 0.001 |
| Employment status |  |  |  |  |
| Employed | Reference |  | Reference |  |
| Housewife | -1.83 (-2.24, -1.43) | < 0.001 | -0.13 (-0.55, 0.32) | 0.560 |
| Unemployed | -3.40 (-3.91, -2.89) | < 0.001 | -0.93 (-1.46, -0.42) | < 0.001 |
| Retired | -2.05 (-2.40, -1.69) | < 0.001 | -0.20 (-0.56, 0.23) | 0.328 |
| Monthly household income, RMB |  |  |  |  |
| ≤ 10,000 | Reference |  | Reference |  |
| > 10,000 | 2.02 (1.71, 2.33) | < 0.001 | 0.25 (-0.01, 0.72) | 0.137 |
| Residence |  |  |  |  |
| Urban | Reference |  | Reference |  |
| Rural/Suburban | -1.43 (-1.76, -1.09) | < 0.001 | -0.03 (-0.33, 0.30) | 0.874 |
| Duration since breast cancer diagnosis |  |  |  |  |
| < 6 months | Reference |  |  |  |
| 6–12 months | 0.28 (-0.33, 0.88) | 0.373 |  |  |
| > 12 months | -0.19 (-0.70, 0.32) | 0.461 |  |  |
| Cancer stage |  |  |  |  |
| Stage Ⅰ | Reference |  | Reference |  |
| Stage Ⅱ | -0.77 (-1.16, -0.38) | < 0.001 | 0.30 (-0.01, 0.72) | 0.106 |
| Stage Ⅲ | -2.42 (-3.00, -1.84) | < 0.001 | -0.48 (-0.98, 0.07) | 0.073 |
| Stage Ⅳ | -2.56 (-4.09, -1.04) | 0.001 | -0.67 (-1.88, 0.68) | 0.306 |
| Unknown | -3.09 (-3.54, -2.64) | < 0.001 | -0.27 (-0.69, 0.22) | 0.249 |
| Treatment completion status |  |  |  |  |
| Not initiated | Reference |  |  |  |
| Under treatment | 0.47 (-0.84, 1.78) | 0.480 |  |  |
| Completed < 1 year ago | 0.17 (-1.31, 1.65) | 0.824 |  |  |
| Completed ≥ 1 year ago | -0.93 (-2.28, 0.42) | 0.177 |  |  |
| Familiarity with telemedicine |  |  |  |  |
| Unfamiliar | Reference |  | Reference |  |
| Familiar | 1.65 (1.33, 1.97) | < 0.001 | 0.17 (-0.16, 0.46) | 0.295 |
| Prior telemedicine usage experience |  |  |  |  |
| No prior experience | Reference |  | Reference |  |
| Has prior experience | 1.74 (1.38, 2.09) | < 0.001 | 0.33 (-0.10, 0.58) | 0.054 |
| Perceived benefits of telemedicine | 2.93 (2.57, 3.30) | < 0.001 | 1.82 (1.45, 2.12) | < 0.001 |
| Perceived risks of telemedicine | -0.69 (-1.11, -0.27) | 0.001 | -0.26 (-0.55, 0.07) | 0.098 |

*: Adjusted for age, marital status, education level, employment status, monthly household income, residence, cancer stage, treatment completion status, familiarity with telemedicine, prior telemedicine usage experience, perceived benefits of telemedicine, and risk concerns of telemedicine.

**Supplementary Table S2.** Sensitivity analysis using a theory-informed multivariable logistic regression model

| Variables | Multivariable analysis* | |
| --- | --- | --- |
|  | OR (95%CI) | P value |
| Age |  |  |
| < 60 | Reference |  |
| ≥ 60 | 0.62 (0.36, 1.08) | 0.094 |
| Marital status |  |  |
| Married | Reference |  |
| Unmarried/Divorced/Separated/Widowed | 0.32 (0.17, 0.59) | < 0.001 |
| Education level |  |  |
| Primary school and below | Reference |  |
| Junior high school | 2.78 (1.70, 4.53) | < 0.001 |
| Senior high school | 2.64 (1.09, 6.40) | 0.031 |
| College | 2.45 (0.90, 6.68) | 0.080 |
| Bachelor’s degree and above | 6.03 (1.60, 22.78) | 0.008 |
| Employment status |  |  |
| Employed | Reference |  |
| Housewife | 0.67 (0.32, 1.43) | 0.298 |
| Unemployed | 0.30 (0.13, 0.70) | 0.005 |
| Retired | 0.60 (0.30, 1.20) | 0.148 |
| Monthly household income, RMB |  |  |
| ≤ 10,000 | Reference |  |
| > 10,000 | 1.35 (0.82, 2.24) | 0.241 |
| Residence |  |  |
| Urban | Reference |  |
| Rural/Suburban | 0.84 (0.51, 1.40) | 0.511 |
| Duration since breast cancer diagnosis |  |  |
| < 6 months | Reference |  |
| 6–12 months | 1.87 (0.85, 4.10) | 0.117 |
| > 12 months | 2.59 (1.27, 5.28) | 0.009 |
| Cancer stage |  |  |
| Stage Ⅰ | Reference |  |
| Stage Ⅱ | 1.25 (0.61, 2.54) | 0.543 |
| Stage Ⅲ | 0.70 (0.30, 1.66) | 0.422 |
| Stage Ⅳ | 0.32 (0.06, 1.82) | 0.200 |
| Unknown | 0.71 (0.33, 1.53) | 0.384 |
| Treatment completion status |  |  |
| Not initiated | Reference |  |
| Under treatment | 1.15 (0.24, 5.44) | 0.859 |
| Completed < 1 year ago | 1.12 (0.19, 6.55) | 0.904 |
| Completed ≥ 1 year ago | 1.20 (0.24, 6.08) | 0.830 |
| Familiarity with telemedicine |  |  |
| Unfamiliar | Reference |  |
| Familiar | 1.11 (0.67, 1.82) | 0.686 |
| Prior telemedicine usage experience |  |  |
| No prior experience | Reference |  |
| Has prior experience | 1.51 (0.82, 2.77) | 0.186 |
| Perceived benefits of telemedicine | 11.98 (5.76, 24.90) | < 0.001 |
| Perceived risks of telemedicine | 0.45 (0.24, 0.84) | 0.012 |

*: Adjusted for age, marital status, education level, employment status, monthly household income, residence, duration since breast cancer diagnosis, cancer stage, treatment completion status, familiarity with telemedicine, prior telemedicine usage experience, perceived benefits of telemedicine, and risk concerns of telemedicine.
